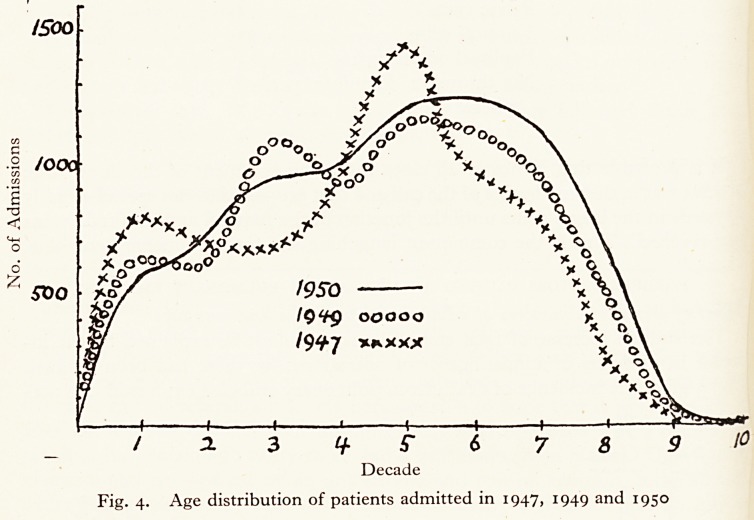# Unexplained Increase of Fatal Pulmonary Embolus at the Bristol Royal Infirmary in 1950

**Published:** 1952-07

**Authors:** Michael G. Wilson

**Affiliations:** Senior Surgical Registrar, Bristol Royal Infirmary


### UNEXPLAINED INCREASE OF FATAL PULMONARY EMBOLUS AT THE BRISTOL ROYAL INFIRMARY IN 1950

BY

MICHAEL G. WILSON, F.R.C.S.

*Senior Surgical Registrar, Bristol Royal Infirmary*

Monthly meetings are held by the staff of the United Bristol Hospitals to discuss recent deaths of patients. At these meetings in 1950 a marked

Fig. 1. Annual deaths from pulmonary embolus, 1940–50

increase in the numbers of deaths from pulmonary embolism was noticed, and it was decided to look further into the matter to find out whether the increase was in fact real, and whether it could be attributed to any particular cause.

Only those cases of pulmonary embolus actually proved by autopsy have been considered, and the inquiry was limited to the Royal Infirmary Branch and therefore includes only medical, surgical, orthopaedic and skin cases.

A study of the annual incidence of fatal pulmonary embolism from 1940 to 1950 showed a gradual slight increase from 1940 to 1949, and then in 1950, a sudden rise to a figure over three times the previous highest (Fig. 1.). In 1950 there were 35 deaths from this cause and in no previous year had there been more than 10.

In the first part of this paper the 1950 cases are analysed in some detail, and in the second part possible causes of the increase are examined.

### ANALYSIS OF THE 1950 CASES

Of the 35 fatal pulmonary emboli, 3 cases may have been pulmonary artery thromboses and in a further 2 the thrombus originated in the auricles of the heart, leaving 30 cases, in 27 of which the source of the embolus was found in the peripheral veins. This analysis applies to the group of 30 cases.

*Age and sex incidence.* More than half of the patients were over 60, and about three-quarters were over 50. Fig. 2 gives the age distribution in decades. There were 17 males and 13 females.

Fig. 2. Age distribution in decades of fatal embolus patients.

*Diagnosis.* It will be seen (Table 1) that half the patients were suffering either from malignant disease or from heart disease. It may be noted here that in the four cases with heart disease a fatal embolus arose in three in the leg and in one only in the auricle. The remainder form a heterogeneous group predominantly of surgical conditions.

TABLE 1

*Underlying disease in 30 cases of fatal pulmonary embolus*

| | | | |
|---|---|---|---|
| Malignant disease .. .. | 11 | D.U. and Pulmonary T.B. | 1 |
| Heart disease .. .. | 4 | D.U. and Hydronephrosis | 1 |
| Acute appendicitis .. | 2 | D.U. .. .. .. | 1 |
| Acute diverticulitis .. | 2 | Strangulated L.I.H. .. | 1 |
| Enlarged prostate .. | 2 | Fractured tib. and fib. .. | 1 |
| Ulcerative colitis .. .. | 1 | Anorexia nervosa .. .. | 1 |
| Acute cholecystitis.. .. | 1 | Cataract .. .. .. | 1 |

*Category.* It was not easy to decide in all cases whether the pulmonary embolus was the sole cause of death. In some it was a terminal event in a patient already moribund. Four categories of cases seemed to emerge. Thus there were seven patients who were already dying, and the embolus was not necessarily a massive one. Four were suffering from incurable disease (e.g. irremovable carcinoma) but they would probably have left hospital alive had they not had a pulmonary embolus. Ten more were suffering from serious conditions but might well have made complete recoveries. Finally nine were suffering from essentially curable conditions and their death was completely unexpected.

In the first two categories the patient's death was perhaps a merciful release, but in the other two groups containing 19 patients it was a catastrophe.

*Operations.* 21 out of 30, or about two-thirds of the patients had had operations of which 7 were for pelvic conditions. In some the operative procedure had been very small, such as the extraction of a cataract or the biopsy of a submental gland. Another patient with an enlarged prostate had been in hospital only three days and was up and about the ward still waiting to have his operation.

*Postoperative day.* In 19 cases in which the embolism followed within two months of operation it occurred as follows:

| *Weeks after operation* | 1 | 2 | 3 | 4 | 5 | 6 | 7 | 8 |
|---|---|---|---|---|---|---|---|---|
| *No. of cases* .. | 6 | 5 | 4 | 1 | 1 | 1 | 0 | 1 |

*Ambulation.* Of the 17 operated cases in which information about ambulation was recorded 9 had been out of bed during the first week after operation.

One of the advantages that was hoped would follow early ambulation was a diminution in the incidence of pulmonary embolism. This does not seem to be the case, and on reflection it seems unreasonable to hope that it would be so—after all, it is not the half-hour up on the day after operation which counts so much as the remaining twenty-three and a half hours spent in bed. One would expect a patient, wearied by an unwelcome and painful exertion, to lie all the stiller on his return to bed. The emphasis surely should be not so much on getting patients up early as on making them move about frequently in bed.

In the "old days" of complete confinement to bed for 10 to 14 days after operation, pulmonary embolus occurred most commonly in the second or third week and was a rare event; now it seems to occur most frequently in the first week after operation and is more common. Out of 6 patients who got their embolus in the first week after operation, 5 were already ambulant, it may well be that early ambulation dislodges some clots which might later become adherent if the major effort of getting out of bed is postponed.

*Intravenous drips.* 11 patients had had fluids administered by intravenous drips, and this included about half the operated cases. Whether the drips were into the arm or into the leg was not recorded, but most patients who have drips for any length of time have them into the leg at some stage. The introduction of foreign bodies and foreign substances into veins, coupled with the immobility of the leg which a drip necessitates, might predispose to thrombus formation: but there was no sudden increase in the use of drips in 1950 to account for the increase in emboli.

*Premonitory signs.* It is too much to expect that a search through patients' notes will give accurate information on this score. However in 6 cases the notes recorded that there were clinical signs in the legs to suggest a deep venous thrombosis. In 11 cases autopsy showed the existence of small recent infarcts in the lungs indicating that the fatal embolus had been preceded by smaller ones. Five patents had received heparin.

*Site of dislodgement of clot.* In all cases except one the venous tree had been carefully opened and searched at post-mortem examination and the sites of dislodgement of clots were found to be as follows:

| | |
|---|---|
| Pelvic veins . . . . | 1 |
| Femoral veins . . . . | 21 |
| Popliteal or calf veins . . | 5 |
| No thrombus found in peripheral veins . . . . | 2 |
| No search made . . | 1 |

It is probable that in nearly all cases the commencement of the thrombotic process was in the small veins of the calf and that propagation clot spread steadily upwards in the larger veins until the junction of the femoral and profunda veins was reached. This is the commonest launching site for a pulmonary embolus.

### POSSIBLE FACTORS CAUSING AN INCREASE IN PULMONARY EMBOLUS

### *Effect of increase in numbers of admissions, operations and autopsies*

The sudden increase of fatal emboli in 1950 cannot be explained by the increase in numbers of admissions or of operations, for these had been gradual. Fig. 3 gives the incidence of death from pulmonary embolus expressed as a per-

Fig. 3. Deaths from pulmonary embolus as a percentage of all post-mortem examinations, and as a percentage of all operations done.

centage of autopsies and of operations—the same sudden increase is observed as in Fig. 1 (annual deaths). It can be seen that in 1950 death occurred from pulmonary embolus in about ten per cent of autopsies and in about 0·44 per cent of patients operated upon. The highest figures previously recorded were 2·7 per cent and 0·23 per cent respectively.

Fatal pulmonary embolus as a percentage of total admissions was 0·15 in 1947, 0·14 in 1949 and 0·5 in 1950—*i.e.* in that year one patient in every 200 admitted died of a pulmonary embolus.

*Effect of increasing age of patients*

There has been an increase in age of patients at the Bristol Royal Infirmary in recent years (Fig. 4). In 1950 there were 156 fewer patients under 50, and 746

Fig. 4. Age distribution of patients admitted in 1947, 1949 and 1950

more over 50 than in 1947 (Table 2). Increasing longevity and greater enterprise in operating on the aged are probably factors in this age shift; but another less obvious cause may be the gradually increasing length of surgical waiting lists. In 1950 there were 1,422 more people waiting for operation than in the corresponding period in 1947. Where there is competition for beds the elderly patients with cancer and serious surgical conditions must displace the younger patients with herniae, varicose veins and conditions which can wait for treatment, and they thus become an undue proportion of the total of admissions. It is in patients over 50 that three-quarters of fatal pulmonary emboli occur.

Nevertheless this shift in age is not the cause of the 1950 increase because the percentage incidence in those over 50 years has doubled while in those under 50 it has increased fourfold compared with 1949 and tenfold compared with 1947 (Table 3).

Thus there has been a real increase in fatal pulmonary embolus in all age groups, proportionately greater in the young than in the old.

TABLE 2

*Age group of in-patients, 1947 and 1950*

| Age Group | Number of in-patients | | | | Increase or Decrease 1947–1950 |
|---|---|---|---|---|---|
| | 1947 | | 1950 | | |
| | Admitted | Percentage of total | Admitted | Percentage of total | |
| Under 50 years | 4,540 | 68·5 | 4,384 | 60·7 | Decrease 156 (7·8%) |
| Over 50 years | 2,090 | 31·5 | 2,836 | 39·3 | Increase 746 (7·8%) |
| Total .. | 6,630 | | 7,220 | | Increase 590 (8·9%) |

TABLE 3

*Incidence of fatal embolus in age groups*

| *Year* | *No. of fatal emboli* | | *No. of patients at risk* | | *Percentage of incidence* | |
|---|---|---|---|---|---|---|
| | Under 50 | Over 50 | Under 50 | Over 50 | Under 50 | Over 50 |
| 1947 .. .. | 1 | 9 | 4540 | 2090 | 0·02 | 0·45 |
| 1949 .. .. | 2 | 8 | 4305 | 2492 | 0·05 | 0·32 |
| 1950 .. | 10 | 25 | 4384 | 2836 | 0·23 | 0·88 |

### EFFECT OF NEW DRUGS AND ANAESTHETIC AGENTS

*Drugs.* The drug schedules of the 1950 patients were searched to see whether any drug was used uncommonly frequently. Out of 30 patients, 19 received penicillin, 15 soneryl, 9 sulphonamides, 8 pethidene, 7 phenobarbitone, 5 streptomycin. Only penicillin and soneryl were used in a significant proportion. The latter drug has been used for many years and can surely be exonerated without further examination.

The case against penicillin has to be considered. The annual issues of penicillin from the dispensary to the hospital wards have been as follows (mega units):

| *1947* | *1948* | *1949* | *1950* |
|---|---|---|---|
| 8,484 | 11,000 | no record | 17,184 |

It is unfortunate that the figures for 1949 are not available, but it is fairly certain that the increase in the use of penicillin has been gradual and that there has been no sudden increase in parallel with the sudden trebling of the incidence of embolus.

Nevetheless there is some evidence to suggest that penicillin increases blood coagulability (Moldavsky *et al.*, 1945) and it may be that the increasing use of penicillin has added something to the general tendency.

*Anaesthetic agents.* The state of complete muscular flaccidity induced by curarine must cause venous stasis in the calves during operations, and might predispose to thrombosis. However, like penicillin, the use of curarine has increased steadily, as the following figures for the issue of ampoules of curarine show.

| *1947* | *1948* | *1949* | *1950* |
|---|---|---|---|
| 750 | 900 | 1,300 | 1,500 |

There therefore seems to be no reason to incriminate this drug.

### INCIDENCE OF FATAL EMBOLUS AT OTHER HOSPITALS

Inquiries have been made from other teaching hospitals about their experience of pulmonary embolus in 1950. Table 4 was compiled from their replies. The

TABLE 4

*Comparison of incidence of pulmonary embolus at various teaching hospitals in* 1950

| Hospital | Admissions | Autopsies | Emboli | Percentage of admissions |
|---|---|---|---|---|
| B.R.I. .. | 7,220 | 341 | 35 | 0·5 |
| A .. .. | 18,073 | 801 | 14 | 0·08 |
| B .. .. | 9,597 | 259 | 4 | 0·04 |
| C .. .. | 8,789 | 197 | 24 | 0·27 |
| D .. .. | 4,934 | 222 | 11 | 0·2 |

figures are not strictly comparable because the type of case dealt with at each hospital differs. Nevertheless, none of these hospitals had noticed a sudden increase in 1950, and nowhere was the incidence more than about half as high as in Bristol.

This suggests that the cause is local to Bristol.

### SUMMARY

1. At the Bristol Royal Infirmary in 1950 there was a remarkable increase in the number of deaths from pulmonary embolus. This was over three times that for any previous year, and was about 0·5 per cent of all admissions. This increase affected all age groups, but was proportionately greater in the young than in the old.

No explanation for this sudden increase was found but the cause is probably local to Bristol and requires further investigation.

2. From 1940 to 1949 there has also been a steady gradual increase in the number of fatal pulmonary emboli. Possible causes for this include:

(*a*) The increasing proportion of hospital patients who are elderly and have serious conditions.

(*b*) The recent tendency towards early ambulation after operations, which probably does not diminish the incidence of phlebo-thrombosis, and may dislodge clots which would otherwise become adherent.

(*c*) The increasing use of penicillin and other antibiotics which may tend to increase blood coagulability.

My thanks are due to the members of the staff of the Bristol Royal Infirmary for allowing me to examine the records of their cases, and to Prof. R. Milnes Walker, Dr. J. E. Cates, Dr. C. S. Shaw and Mr. A. Levy for valuable help.

*Addendum*

1951 figures are now available and show that the increase has been maintained; the number of deaths from pulmonary embolus was 31, compared with 10 in 1949, and 35 in 1950.

### REFERENCE

Moldavsky, L. F., Hasselbrock, W. B., Cateno, C., *Science*, 1945, **102**, 38.

## Figures and Tables

**Fig. 1. f1:**
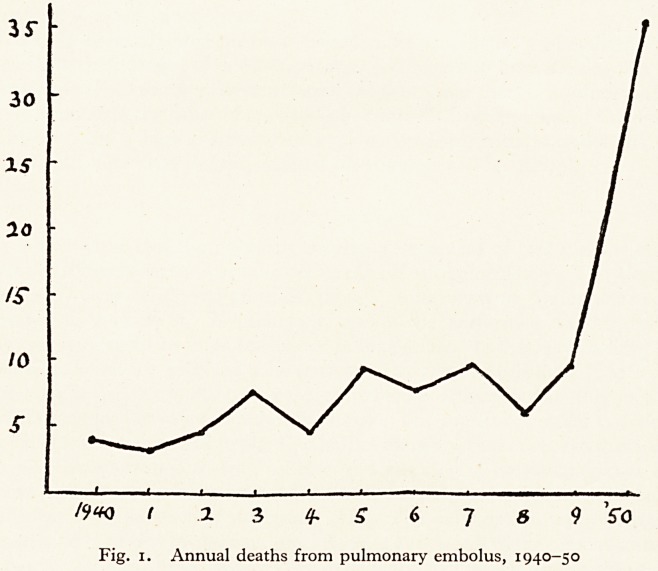


**Fig. 2. f2:**
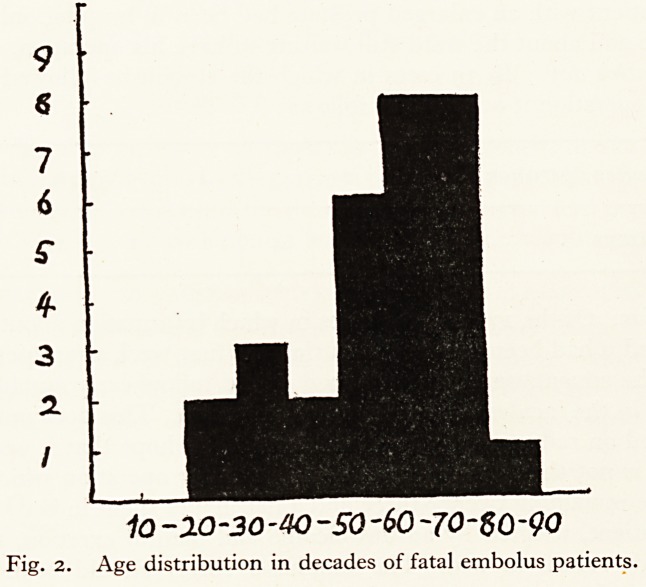


**Fig. 3. f3:**
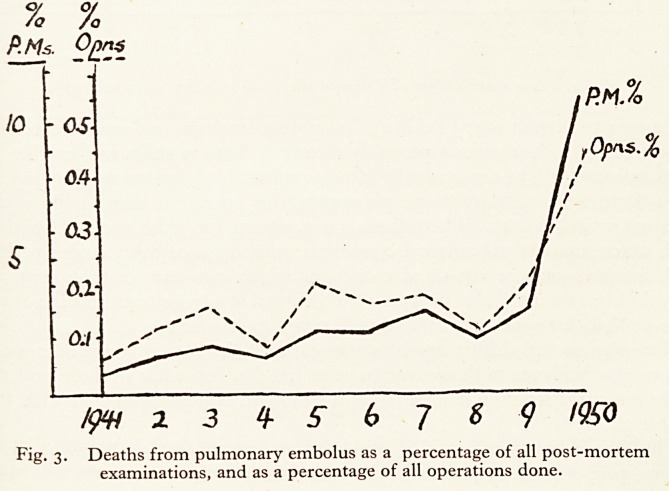


**Fig. 4. f4:**